# Can lettuce plants grow in saline soils supplemented with biochar?

**DOI:** 10.1016/j.heliyon.2024.e26526

**Published:** 2024-02-15

**Authors:** Riccardo Fedeli, Andrea Vannini, Nesrine Djatouf, Silvia Celletti, Stefano Loppi

**Affiliations:** a Department of Life Sciences, University of Siena, 53100, Siena, Italy; b BAT Center - Interuniversity Center for Studies on Bioinspired Agro-Environmental Technology, University of Naples “Federico II”, 80138, Napoli, Italy

**Keywords:** Antioxidants, Charcoal, Electrolyte leakage, Free amino acids, Salinity stress

## Abstract

Salt stress is presently a major environmental concern, given the huge number of soils affected by the presence of dissolved salts. Therefore, it is necessary to find solutions, preferably nature-based ones, to deal with this problem. In this study, biochar, a product made from plant biomass residues through the process of pyrolysis, was tested to alleviate salt stress on lettuce (*Lactuca sativa* L.) plants. Six different concentrations of NaCl were tested: 0, 50, 100, 200, 300 and 400 mM with and without the addition of 5% (w/w) biochar. Biochar ability to mitigate salinity damage was assessed by means of both biometric (fresh weight), physiological (chlorophyll content), and biochemical (i.e., electrolyte leakage, total antioxidant power, total soluble proteins, free amino acids, and mineral content) parameters. The experiment lasted four weeks. The results showed that NaCl has a negative effect from the concentration of 100–200 mM and that biochar was to some extent effective in mitigating the negative effects of salt on plant physiology; nevertheless, biochar failed to counteract Na accumulation. Similarly, biochar did not influence the content of free amino acids in lettuce leaves, but enhanced the expression of several parameters, such as total antioxidant power, fresh weight, chlorophyll content, total soluble protein, K content, although only clearly evident in some cases. Overall, the present study showed that biochar is a viable solution to counteract the damage caused by high salt concentrations on plant growth.

## 1 Introduction

Climate change is the major environmental concern worldwide [1,2], among others, one consequence of climate change is the increase in the content of soluble salts (such as sodium calcium and magnesium sulfates, chlorides, and bicarbonates) in the soil, often to an extent that crop species are negatively affected, and in this case, the soil is classified as “saline”. Sodium (Na) is the most harmful salt, causing shriveled foliage, hindered plant development, and dry, discolored plant tissues, since it ruins plant water retention [3]. Moreover, Na can cause toxicity and react with plant tissues, creating an effect called osmotic stress [3]. This phenomenon is particularly relevant in arid and semi-arid areas (e.g., Asia, Australia, Africa, and South America), where, due to the high evapotranspiration, water transports salts to the soil surface [[4], [5], [6]]. Soil salinization is one of the major soil degradation threats in Europe as well, being estimated to affect 1–3 million ha, mainly in the Mediterranean countries [7]. Soil salinization is included among the nine major issues indicated by Food and Agriculture Organization (FAO) and it should not be neglected as it dramatically impacts the ecological functions of soil, and hence agriculture [8].

The excess Na in the soil can displace other essential nutrients such as Ca, Mg, and K, which are critical for plant growth and development, leading to nutrient deficiencies in plants and reduced crop yield [9]. As early as 2008, the problem of salinization plagued more than 1 billion ha of arable soil globally [10], and, unfortunately, this value has not stopped, reaching 1.2 billion ha in 2017, due to wrong agricultural soil management practices pursued for years and exacerbated by the effect of ongoing climate change [11]. At a global scale, the maximum levels of salinity observed in soils can vary depending on the specific geographical region and local conditions (i.e., Death Valley, USA: 270 dS m^−1^; Iran: 200 dS m^−1^, Central Asia: 100 dS m^−1^) [12].

The increase in saline soils consequently causes substantial crop yield losses. As an example, due to high concentration of salts in the soil, the average crop yield loss in one year has been estimated to be 30% [13] and 25% [14] in Sindh (Pakistan, South Asia) and Alberta (Canada, North America), respectively. Yield losses of this magnitude severely affect farmers’ income, generating on average economic losses of about $27.3 billion per year globally [15]. Overall, therefore, considering that around 10 billion people will need to be fed in 2050 and that global agricultural productivity will need to be increased by about 1.75% per year to meet this exponential population growth [16], the efforts required in looking for approaches that, on the one hand, are not a threat to the environment, and, on the other hand, are efficient in limiting the loss of arable soil are not trivial.

There are several strategies to mitigate salt stress. Leaching is one of the most effective methods of removing salts from the root zone of soils and is achieved by accumulating fresh water on the soil surface and allowing it to infiltrate. Flushing, which involves washing away accumulated salts at the surface by running water over the surface, is sometimes used to desalinate soils that have surface salt crusts, but this method is of little practical importance because the quantity of salts that can be washed away from a soil is quite small [11]. Phytoremediation is another method that can be cost-effective and eco-friendly, but it is not as effective as leaching [17]. Agronomic practices, such as using salt-tolerant crop varieties, reducing tillage, and adopting crops with low water requirements, can be used to recover saline soils [17]. In recent times, a novel product has been proposed as a potential countermeasure to mitigate the detrimental impact of salt, namely, biochar [18].

Biochar is a solid product derived from organic material, such as plant biomass (essentially tree crop pruning and agro-industrial residues), subjected to the process of non-oxidative thermal decomposition (known as pyrolysis) [19,20]. Biochar is a carbon-rich material and therefore, when incorporated into the soil, it plays a remarkable environmental function as it contributes to the sequestration of atmospheric carbon in the soil and improves many physical (porosity and aeration), chemical (cation exchange capacity) and biological properties (microbiological activity) of the soil [[21], [22], [23], [24]]. Biochar has also proved to be a very effective component of the growing medium as a substitute for peat for plant growth [25,26]. However, due to its chemical characteristics, mainly related to its high salt content and pH, the recommended dose of biochar in the soil should not exceed 15% (w/w), otherwise possible undesirable effects on plant growth and development could appear [27]. In addition, biochar can retain potentially toxic elements (such as Pb and Cd) in the soil, as well as to reduce the availability of hydrocarbons and salts to plants [[28], [29], [30]], hence increasing crop productivity and the quality of agricultural products [31,32]. Specifically, the ability of biochar to enhance plant growth when subjected to salt stress conditions has been documented in previous studies conducted on tomato (*Solanum lycopersicum* L.) [33], corn (*Zea mays* L.) [34], and eggplant (*Solanum melongena* L.) [35]; moreover, the use of biochar exhibited a notable 36% decrease in electrolyte (EL) in salt-stressed kochia (*Bassia scoparia* L.) plants [36]. For all these reasons, in Italy, biochar is included among the soil improvers that can be used not only in conventional agriculture [37], but very recently also in organic farming [38].

Lettuce (*Lactuca sativa* L.) is one of the most widely cultivated and consumed food plant species in the world, especially in the Americas, Europe, and China [39], due to of high nutritional value of its leaves containing a high content of active biomolecules, such as vitamins (A and B6) and polyphenols, important for human health, protecting the immune system, controlling the nervous system, and regulating the metabolism [40,41]. Like most crop plants, lettuce is moderately tolerant to soil salinity and can show symptoms of toxicity at concentrations above 100–200 mM Na chloride (NaCl) [42]. The negative effects can be seen at any phenological stage, (starting from seed germination), causing initially an impairment of the photosynthetic process, a reduction of the leaf and root growth and leaf wilting, which if the stress period is prolonged can lead to plant death, with consequent negative effects on the nutritional profile of the edible product [[43], [44], [45]]. On the other hand, it has also been shown that providing moderate salt concentrations (around 50 mM) can improve lettuce quality and yield [46].

Since soil salinity is an environmental concern which accounts for 20% of the world's crop production loss [47], it is of utmost importance to act in finding environmentally sustainable solutions to limit the harmful consequences of this phenomenon on crops. In this context, this work aimed to verify whether the addition of 5% (w/w) biochar to the soil could alleviate the effects of NaCl added to the soil at different concentrations (0, 50, 100, 200, 300, and 400 mM) on the physiological (total chlorophyll content), biometric (leaf fresh biomass), and biochemical (electrolyte leakage, total antioxidant power, total soluble proteins, free amino acids, and mineral element content) parameters of lettuce plants.

## 2 Materials and methods

### 2.1 Biochar

Biochar (B) was provided by a local enterprise (BioEsperia, Arezzo, Italy) [48], and obtained through a pyrolysis process at a temperature of 600–650 °C. The feedstock was composed of a mixture of organic waste of agricultural and forestry origin such as olive pomace, grape marc, walnut shells, and wood from tree prunings. Table 1 shows the physico-chemical characteristics of the B used in the experiment.

**Table 1:** Physico-chemical characteristics of biochar.

Particle diameter (mm)	<0.5
WHC (%)	23.5
EC (mS m^−1^)	110.0
pH	9.9
Hash content (%)	7.0
H/C	0.2
Total N (%)	<0.5
Total P (mg kg^−1^)	340.0
Total K (mg kg^−1^)	3.0
Total C (mg kg^−1^)	9.9
Total Mg (mg kg^−1^)	852.0
Total Na (mg kg^−1^)	291.0
C from carbonate (%)	<0.1

WHC: water holding capacity; EC: electrical conductivity; H: hydrogen.

C: carbon; N: nitrogen; P: phosphorous; K: potassium; Mg: magnesium; Na: sodium.

### 2.2 Plant growth and treatments

Lettuce seedlings (*Lactuca sativa* L., cv. Salanova) were purchased from a local nursery (Siena, Tuscany, Italy). Once in our laboratory, the seedlings were removed from their containers and transplanted (1 seedling/pot) into black plastic pots (10 × 10 × 10 cm), containing soil previously sieved to < 2 mm. A total of 60 pots was prepared, 30 of which containing only soil (B0), and the remaining 30 containing soil with the addition of 5% (w/w) biochar (B5). Immediately after transplanting, the seedlings were watered with the following NaCl solutions (JT Baker – Fisher Scientific, Milan, Italy), dissolved in deionized water, at six different concentrations: 0, 50, 100, 200, 300 and 400 mM. Five replicates were prepared for each treatment. Plants were grown for four weeks in a climate chamber at 23 °C, 60% relative humidity and 220 μmol m^−2^ s^−1^ PAR light intensity with a 16/8 h day/night cycle. During the experimental growth period, plants were watered once a week with the different NaCl solutions, by maintaining the field capacity of the soil in each pot constantly at 60%. At the end of the 4-week growth period, only the above-ground part of lettuce plants was harvested, weighed in terms of leaf fresh biomass, and stored at −20 °C for the subsequent physiological and biochemical analyses.

### 2.3 Leaf analyses

#### 2.3.1 Total chlorophyll

The total chlorophyll content on a surface basis (mg m^−2^) was estimated in the attached leaves of lettuce plants with a portable and non-destructive chlorophyll content meter (CCM – 300, Opti-Sciences Inc., Hudson, NH, USA). Three measurements (one distal, one central, and one proximal from the leaf apex) per leaf were recorded on each plant, using the three leaves fully expanded from the top of the plant.

#### 2.3.2 Electrolyte leakage

Electrolyte leakage from leaf tissue was determined following the method described by Dionisio-Sese and Tobita [49] and Sunkar et al. [50], with slight modifications. The youngest fresh leaves of lettuce plants were washed thoroughly with distilled water (dH_2_O). From them, pieces of 200 mg of uniform size (5 mm × 5 mm) were cut and immersed in 20 mL of dH_2_O for 2 h at room temperature. Subsequently, the electrical conductivity (EC1) of the solutions containing the different samples was measured with a conductivity-meter (BASIC 30, Crison Strumenti SpA, Carpi, MO, Italia). Finally, these solutions were heated for 25 min at 90 °C and then returned to room temperature before EC (EC2) was measured again. The EC of the different leaf samples was expressed as a percentage and calculated as follows:

(1) $$EL\;\left(\%\;\right)=\left(\frac{EC1}{EC2}\right)\times 100$$

#### 2.3.3 Total antioxidant power

The total antioxidant power of lettuce leaves was established according to Loppi et al. [51], with slight modifications. Frozen leaf material (500 mg) was homogenized in 2 mL of 80% (v/v) ethanol for 2 min and centrifuged at 15000 rpm for 5 min. A 200 μL aliquot of the supernatant recovered from each sample was added to 1 mL of 2,2-Diphenyl-1-picrylhydrazyl (DPPH) solution (obtained by dissolving 3.9 mg DPPH in 100 mL of 80% (v/v) methanol). A blank and a control were prepared by adding 200 μL of 80% (v/v) ethanol into 1 mL of 80% (v/v) methanol and 1 mL of the DPPH solution, respectively. After 1 h incubation in the dark, the absorbance of the samples was measured at 517 nm by means of a UV–Vis spectrophotometer (8453, Agilent, Santa Clara, CA, USA). Results were expressed as the percentage of anti-radical activity (ARA, %) according to the following formula:

(2) $$ARA\;\left(\%\right)=100\times \left(1-\frac{sample\;absorbance}{control\;absorbance}\right)$$

#### 2.3.4 Total soluble proteins

The total soluble protein content of lettuce leaves was measured following the method of Celletti et al. [52]. Briefly, frozen leaf samples (0.250 g) were homogenized in 1.5 mL of dH_2_O for 2 min and centrifuged at 3000 rpm for 5 min. The recovered supernatants were centrifuged at 12000 rpm for 7 min. Subsequently, the newly recovered supernatants were diluted 1:50 with dH_2_O (v:v) and 0.4 mL of the diluted samples was added to 1.6 mL of Bradford's reagent solution (Thermo Fisher Scientific Inc., Waltham, MA, USA. The samples were allowed to react for 20 min at room temperature and then their absorbance was recorded at 595 nm using a UV–Vis spectrophotometer (8453, Agilent, Santa Clara, CA, USA). Protein concentrations in the samples were finally calculated using a calibration curve obtained with bovine serum albumin (BSA) (Sigma-Aldrich, USA) used as a standard.

#### 2.3.5 Free amino acids

Free amino acids (FAAs) in the leaves of lettuce plants were determined following the method reported by Fedeli et al. [53]. Fresh samples (about 1 g) were homogenized in 2 mL dH_2_O for 2 min and then centrifuged at 4000 rpm. According to the AccQ Tag protocol (Waters, Milford, MA, USA), 10 μL of each reconstituted sample was derivatized with amino acids [54], using the fluorescent reagent AQC (Waters, Milford, MA, USA) and 0.02 M borate buffer (pH 8.6). FAAs were separated and quantified using a high-pressure liquid chromatography system (HPLC- LC1, Waters, Milford, MA, USA) equipped with an C18 column (250 × 4.6 mm, 5 μm, Agilent, Santa Clara, CA, USA), thermostated at 20 °C, and a scanning fluorescence detector (470, Waters, Milford, MA, USA), (excitation at 250 nm, detection at 395 nm). The solvents used were: (A) 22.9% (w/v) sodium acetate/water, 7.7% (v/v) phosphoric acid/water and 4.1% (v/v) triethylamine/water; (B) 60% (v/v) acetonitrile/deionized water. The concentration of each amino acid (alanine - Ala), (arginine - Arg), (aspartic acid - Asp), (glutamic acid - Glu), (glycine - Gly), (histidine - Hys), (leucine - Leu), (lysine - Lys), (methionine - Met), (proline - Pro), (serine - Ser), (threonine - Thr), (tyrosine - Tyr), and (valine - Val) was estimated by matching the area under the peak of the chromatogram to the standard (WAT088122, Waters, Milford, MA, USA), using Clarity software (DataApex).

#### 2.3.6 Mineral elements

To quantify the content of mineral elements [(calcium (Ca), magnesium (Mg), potassium (K), phosphorus (P), copper (Cu), iron (Fe), manganese (Mn), zinc (Zn), and sodium (Na)], lettuce leaves were oven-dried at 60 °C to constant weight and then pulverized with mortar and pestle. Subsequently, 0.150 g_DW_ of sample was mineralized with 3 mL of 70% (v/v) HNO_3_, 0.2 mL of 50% (v/v) HF and 0.5 mL of 30% (v/v) H_2_O_2_, using a microwave digestion system (Milestone Ethos 900, Metrohm, Australia) at 280 °C and 55 bar, following the method reported in Fedeli et al. [55]. The elemental content was determined by inductively coupled plasma – mass spectrometry (ICP – MS, PerkinElmer NexION 350, MA, USA). Analytical quality was checked using the NCS DC 73350 certified standard reference material “*Poplar leaves*”; recoveries ranged 96–111%. The precision of the analysis was estimated by the coefficient of variation of five biological replicates and was always >97%.

### 2.4 Statistical analysis

Since the data did not approach a normal distribution (Shapiro-Wilk test, *p* ≤ 0.05), non-parametric tests were used [56]. Parameter estimates were expressed by their median value and the associated error by the interquartile range divided by the square root of the number of observations from five biological replicates (n = 5). The significance of differences (*p* ≤ 0.05) between treatments was assessed by Kruskal-Wallis ANOVA, while the Dunn's test was used for pairwise comparisons. All calculations and graphs were made with the R free software [57].

## 3 Results

**Fig. 1:**
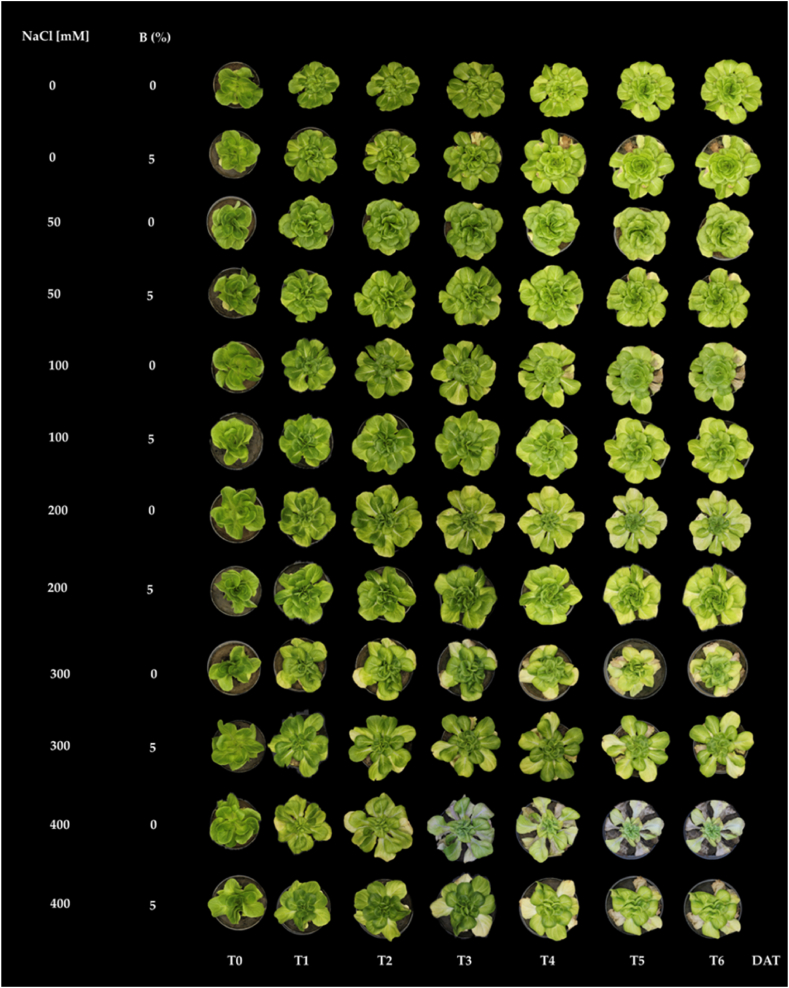
Phenotypic time changes in lettuce plants subjected to grow for four weeks under different concentrations (0–400 mM) of NaCl with or without biochar (B) mixed with the soil at 5% (w/w). T0 = 0, T1 = 8, T2 = 10, T3 = 14, T4 = 16, T5 = 21, T6 = 27 days after transplanting (DAT).

Fig. 1 indicates that up to a concentration of 200 mM NaCl no signs of salt damage on lettuce plants were visible at any time, whereas, at concentrations of 300 and 400 mM NaCl, signs of damage could be observed starting from T3. Notably, the addition of biochar seems to mitigate the deleterious effects of salt stress, resulting in improved plant growth and vigor.

### 3.1 Effects of NaCl addition to the soil

**Fig. 2:**
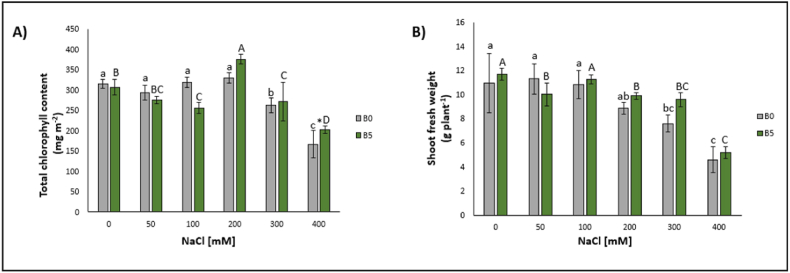
Chlorophyll content (A) and shoot fresh weight (B) of lettuce leaves expressed as median ± error. The number along the horizontal axes indicates the concentration of NaCl added to the growing medium. B0 = without biochar; B5 = with 5% (w/w) biochar. Different letters (lowercase for B0 and uppercase for B5) indicate significant differences between the same treatment; symbol * indicates the significant differences (p ≤ 0.05) in pairs relative to the same treatment.

Treated lettuce plants showed statistically significant decreases in chlorophyll content at NaCl exposures of 300 mM (−17%) and 400 mM (−47%) (Fig. 2A).

Similarly, plants fresh weight experienced reductions following the exposure of lettuce to concentrations of 300 mM (−31%) and 400 (−66%) mM (Fig. 2B).

**Fig. 3:**
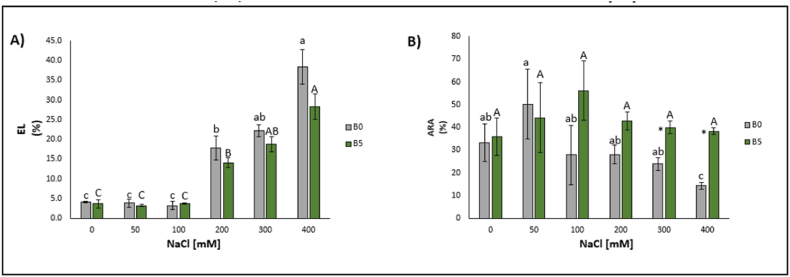
Electrolyte leakage (EL) (A) and anti-radical activity (ARA) (B) of lettuce leaves expressed as median ± error. The number along the horizontal axes indicates the concentration of NaCl added to the growing medium. B0 = without biochar; B5 = with 5% (w/w) biochar. Different letters (lowercase for B0 and uppercase for B5) indicate significant differences between the same treatment; symbol * indicates the significant differences (p ≤ 0.05) in pairs relative to the same treatment.

Concerning the electrolyte leakage, statistically significant differences were observed for lettuce plants exposed to 200 mM (+336%), 300 mM (+441%), and 400 mM NaCl (+837%) concentrations (Fig. 3A).

Total antioxidant power showed a statistically significant reduction only following the highest NaCl treatment concentration (400 mM; −57%) (Fig. 3B).

**Fig. 4:**
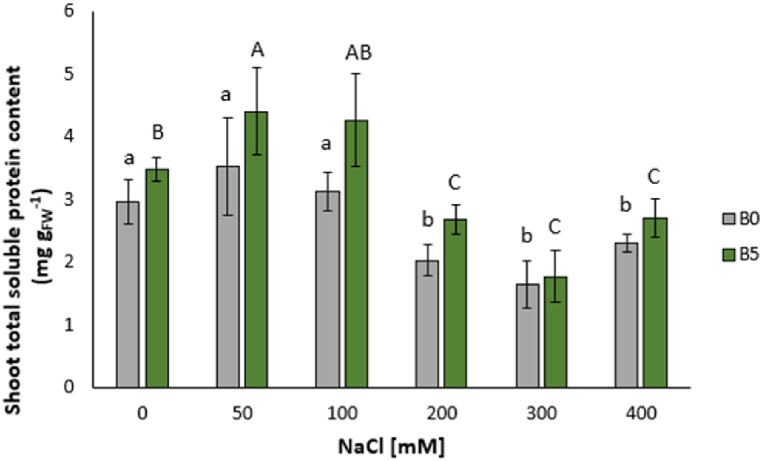
Shoot total soluble protein content of lettuce leaves expressed as median ± error. The number along the horizontal axes indicates the concentration of NaCl added to the growing medium. B0 = without biochar; B5 = with 5% (w/w) biochar. Different letters (lowercase for B0 and uppercase for B5) indicate significant differences between the same treatment; symbol * indicates the significant differences (p ≤ 0.05) in pairs relative to the same treatment.

Total soluble proteins content showed significant decreases for plants treated with 200 mM (−32%), 300 mM (−45%), and 400 mM NaCl (−23%) (Fig. 4).

**Fig. 5:**
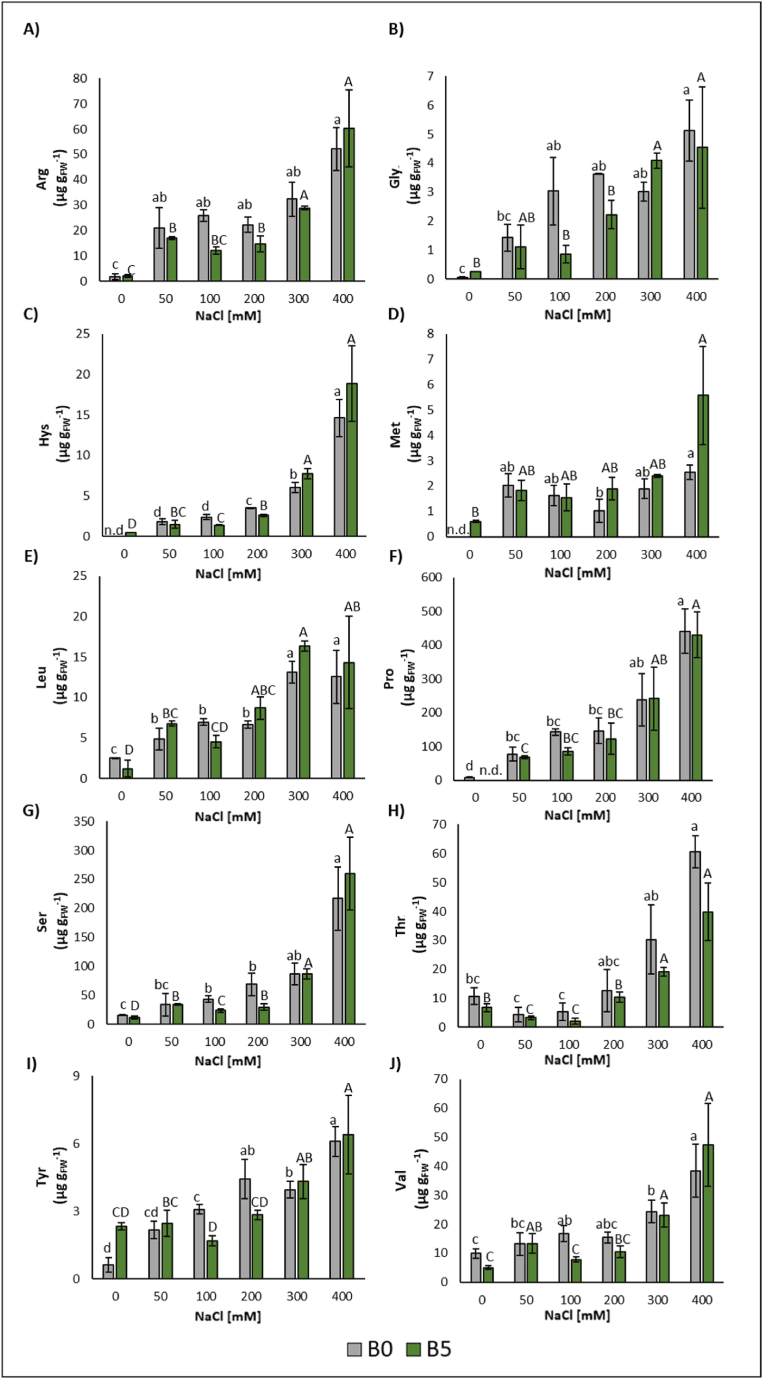
Arginine (Arg) (A), glycine (Gly) (B), histidine (Hys) (C), methionine (Met) (D), leucine (Leu) (E), proline (Pro) (F), serine (Ser) (G), threonine (Thr) (H), tyrosine (Tyr) (I), valine (Val) (J) content of lettuce leaves expressed as median ± error. The number along the horizontal axes indicates the concentration of NaCl added to the growing me-dium. B0 = without biochar; B5 = with 5% (w/w) biochar. Different letters (lowercase for B0 and upper-case for B5) indicate significant differences (p ≤ 0.05) between the same treatment. n.d. = not determined.

Regarding the content of FAAs, with the exception of Asp there was a significant increase in the various treatments (Fig. 5A–J, Table S1). Hys, Arg, Pro, Leu, and Met showed a significant increase in content from the concentration of 50 mM NaCl reaching a maximum value at 400 mM NaCl of: 1463-fold, 29-fold, 53-fold, 4-fold, 254-fold, respectively. Ser, Gly, and Tyr showed a significant increase in content from the concentration of 100 mM NaCl reaching a maximum value of: +1290%, +8433%, and +913% at the concentration of 400 mM, respectively. Glu showed a significant increase at the concentration of 100 mM NaCl (+97%) and 400 mM (+134%). Ala showed a significant increase at the concentration of 200 mM NaCl (+185%). Thr showed a significant increase at the concentration of 400 mM NaCl (+470%). Val showed a significant increase at the concentrations of 100, 300, and 400 mM NaCl (+68%, +146%, +287%, respectively). Lys showed a significant increase at the concentration of 300 mM NaCl (+479%).

**Fig. 6:**
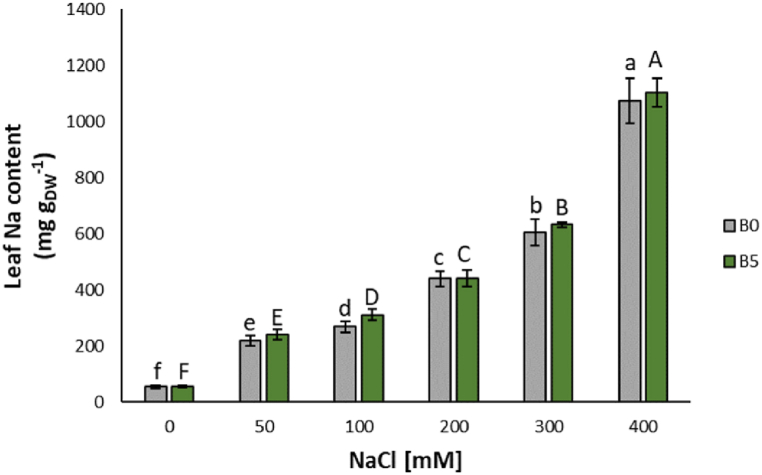
Sodium (Na) content of lettuce leaves expressed as median ± error. The number along the horizontal axes indicates the concentration of NaCl added to the growing medium. B0 = without biochar; B5 = with 5% (w/w) biochar. Different letters (lowercase for B0 and uppercase for B5) indicate significant differences (p ≤ 0.05) between the same treatment.

The concentration of Na in lettuce leaves showed statistically significant increases in proportion to the tested NaCl treatment concentrations: 50 mM (+295%), 100 mM (+342%), 200 mM (+683%), 300 mM (+918%), and 400 mM (+1690%) (Fig. 6).

The content of K showed statistically significant reductions following the treatment with 100 mM (−29%), 200 mM (−67%), 300 mM (−61%), and 400 mM NaCl (−61%); the content of Zn and Cu showed statistically significant reductions as a consequence of the treatment with 200 mM (−41%; −48%), 300 mM (−35%; −37%), 400 mM NaCl (−23%; −46%, respectively); Ca and P showed statistically significant reductions in the contents following the treatment with 200 mM NaCl (−42%; −35%; respectively); Mn, instead, showed a statistically significant increase at 100 mM (+57%) and 300 mM NaCl (+11%) (Table 2).

**Table 2:** Content of mineral elements of lettuce leaves expressed as median ± error. B0 = without biochar; B5 = with 5% (w/w) biochar. Ca: calcium; Mg: magnesium; K: potassium; P: phosphorus; Cu: copper; Fe: iron; Mn: manganese; Zn: zinc. Different letters (lowercase for B0 and uppercase for B5) indicate significant differences between the same treatment; where letters are not present, there is no statistical difference; symbol * indicates the significant differences (p ≤ 0.05) in pairs relative to the same treatment.

**B (%)**	**NaCl [mM]**	**Ca**	**Mg**	**K**	**P**
		**(mg g**_**DW**_^**−1**^**)**			
**0**	0	21.5 ± 3.61^a^	3.07 ± 0.64	44.4 ± 6.76^a^	3.06 ± 0.02^a^
**5**		18.6 ± 1.51^A^	2.51 ± 0.11	54.9 ± 1.91^A^	3.06 ± 0.45^A^
**0**	50	16.2 ± 1.34^a^	2.56 ± 0.34	37.2 ± 5.65^a^	3.52 ± 0.32^a^
**5**		15.5 ± 1.31^A^	2.74 ± 0.15	32.0 ± 5.53^AB^	2.92 ± 0.26^AB^
**0**	100	14.4 ± 2.19^ab^	2.76 ± 0.16	31.5 ± 2.17^b^	2.69 ± 0.052^a^
**5**		15.6 ± 1.51^AB^	2.84 ± 0.074	31.1 ± 4.94^BC^	2.46 ± 0.15^C^
**0**	200	12.5 ± 0.35^b^	2.31 ± 0.13	14.6 ± 1.65^c^	1.99 ± 0.22^b^
**5**		13.6 ± 1.01^AB^	2.80 ± 0.27	26.0 ± 0.81^BC^*	2.50 ± 0.15^BC^
**0**	300	13.3 ± 0.85^ab^	2.75 ± 0.18	17.3 ± 1.32^c^	2.46 ± 0.071^ab^
**5**		11.5 ± 1.01^B^	2.44 ± 0.27	19.2 ± 0.47^D^	2.33 ± 0.13^C^
**0**	400	16.6 ± 0.81^a^	2.45 ± 0.10	17.5 ± 0.84^c^	3.39 ± 0.15^a^
**5**		14.3 ± 0.85^AB^	2.92 ± 0.20	22.7 ± 0.22^CD^*	3.07 ± 0.19^A^
		**Cu**	**Fe**	**Mn**	**Zn**
		**(mg g**_**DW**_^**−**^**^1^)**			
**0**	0	0.0075 ± 0.0004^a^	0.1.09 ± 0.06	2.22 ± 0.59bc	0.054 ± 0.011^a^
**5**		0.0081 ± 0.0006^A^	0.074 ± 0.021^B^	0.25 ± 0.038^BC^	0.039 ± 0.0043^A^
**0**	50	0.0072 ± 0.0009^a^	0.11 ± 0.019	0.30 ± 0.026^ab^	0.041 ± 0.0031^a^
**5**		0.0071 ± 0.0006^A^	0.11 ± 0.025^B^	0.30 ± 0.017^AB^	0.046 ± 0.012^AB^
**0**	100	0.0066 ± 0.0004^a^	0.088 ± 0.032	0.34 ± 0.072^a^	0.036 ± 0.0081^ab^
**5**		0.0056 ± 0.0004^B^	0.11 ± 0.0072^B^	0.32 ± 0.032^A^	0.034 ± 0.0031^CD^
**0**	200	0.0044 ± 0.0004^b^	0.10 ± 0.0083	0.26 ± 0.041^bc^	0.029 ± 0.0009^c^
**5**		0.0051 ± 0.0002^BC^	0.095 ± 0.0095^B^	0.28 ± 0.047^AB^	0.039 ± 0.0036^BC^
**0**	300	0.0048 ± 0.0001^b^	0.14 ± 0.035	0.24 ± 0.027^c^	0.034 ± 0.0042^bc^
**5**		0.0047 ± 0.0003^C^	0.10 ± 0.011^B^	0.15 ± 0.027^C^	0.021 ± 0.0007^D^
**0**	400	0.0057 ± 0.0005^b^	0.22 ± 0.045	0.21 ± 0.011^c^	0.029 ± 0.0003^c^
**5**		0.0055 ± 0.0004^BC^	0.20 ± 0.039^A^	0.16 ± 0.008^C^	0.041 ± 0.0024*^ABC^

### 3.2 Effects of NaCl addition to the soil conditioned with biochar

The addition of 5% (w/w) of B increased in the content of chlorophylls at 200 mM (+15%) and a decrease at the 100 mM (−13%), 300 mM (−14%), and 400 mM NaCl (−36%) concentrations (Fig. 2A).

Fresh weight of the lettuce plants experienced decreases at the 50 mM (−14.53%), 200 mM (−15%), 300 mM NaCl (−18%), and 400 mM NaCl (−57%) (Fig. 2B).

Regarding the electrolyte leakage, statistically significant increases were observed following the treatment with 50 mM (−20%), 200 mM (+235%), 300 mM (+359%), and 400 mM NaCl (+590%) (Fig. 3A).

No statically significant differences were observed with the addition of 5% (w/w) of B in the DPPH content (Fig. 3B).

The content of total soluble proteins experienced significant increases following the treatment with 50 mM (+48%) and 100 mM NaCl (+44%) and a decrease as a consequence of that at 200 mM (−21%), 300 mM (−41%), and 400 mM (−23%) (Fig. 4).

Concerning the content of FAAs, and except for Asp, there was a significant increase in the various treatments where biochar was added (Fig. 5A–J, Table S1). His, Arg, Pro, and Ser showed a significant increase from the concentration of 50 mM NaCl, reaching a maximum at 400 mM of 1884-fold, 34-fold, 43045-fold,16-fold, respectively. Glu showed a significant increase at 400 mM (+134%). Gly showed a significant increase at 300 mM and 400 mM (15-fold, 16-fold). Thr showed a significant decrease at 50 and 100 mM (−52%, −69%) and a significant increase at 300 and 400 mM (+182%, +490%, respectively). Val showed a significant increase at 50 mM, 300 mM and 400 mM (+165%, +359%, +844 respectively). Leu showed a significant increased for all the concentrations, expect 100 mM (+449%, +602%, +1221%, +1054%). Met showed a significant increase at 400 mM (+830.02%). Thr showed a significant decreased at 50 mM and 100 mM (−51%, −68%) and a significant increase at 300 and 400 mM (+190%, +506%). Lys showed a significant increase at 300 mM (+496%).

The content of Na in lettuce leaves showed statistically significant increases proportionally to NaCl concentrations: 50 mM (+292%), 100 mM (+437%), 200 mM (+676%), 300 mM (+985%), and 400 mM (18-fold) (Fig. 6).

Concerning the content of chemical elements (Table 2), treated plants showed statistically significant reductions in the content of K and Cu following exposure to 100 mM (−30%; −25%), 200 mM (−42%; - 33%), 300 mM (−57%; −36%), and 400 mM NaCl (−49%; −27%), in the content of Zn and P following exposure to 100 mM (−38.89%; −19.50%), 200 mM (−29%; −18%), and 300 mM NaCl (−61%; −24%), as well as in the content of Ca following exposure to 300 mM NaCl (−47%). Additionally, treated plants showed statistically significant increases in Mn at 100 mM NaCl (+49%) and Fe after exposure to 400 mM NaCl (+175%).

### 3.3 Effects of biochar conditioning

Chlorophyll content showed a significant increase at the 400 mM (+21%) (Fig. 2A).

The plant fresh weight did not show any significant difference, but there was an increasing trend from the concentration of 200 mM (Fig. 2B).

As for electrolyte leakage, no significant difference was evident, but a decreasing trend was well observed starting from the concentration of 200 mM (Fig. 3A).

The total antioxidant power increased significantly at 300 mM (+67%) and 400 mM (+167%) (Fig. 3B).

No significative differences in FAAs were found following the addition of biochar (Fig. 5A–J, Table S1).

All nutrients did not show any statistical differences, but K and Zn (Fig. 6, Table 2); K showed an increase at 200 mM (+78%) and 400 mM (+30%), while Zn only at 400 mM (+43%) (Table 2).

## 4 Discussion

### 4.1 Response to salt stress

There are reports in literature that lettuce plants experience growth problems from concentrations of 100–200 mM NaCl in the soil [42], and our results are consistent with this view since for most parameters the negative effect of salt becomes evident from the concentration of 200 mM NaCl. Below this concentration, several studies report that the presence of NaCl succeeds in promoting plant growth by acting as a stimulant, shifting plant metabolism to a buildup of antioxidant molecules that are not only important for the defence against various stresses, but have also a beneficial role for human health after regular consumption [34,58,59]. Our findings are consistent with the above-mentioned studies since biometric, photosynthetic, and biochemical parameters did not decrease, but in some cases, e.g., for DPPH and total soluble proteins, even increased. Santander et al. [60] and Borgognone et al. [61] showed that lettuce plants treated with moderate concentrations of NaCl (50 and 40 mM, respectively) increased the content of antioxidant metabolites (*i.e.*, phenols, luteolin, cynarine, and chlorogenic acid) and total antioxidant power without going to affect the growth and the biomass of lettuce plants. Kim et al. [34] also showed that treatment with a concentration of 50 mM NaCl increased the total phenolic content without adversely affecting plant fresh weight and the visual quality of leaves. Conversely, when higher NaCl concentrations (150–200 mM NaCl) are added, the total antioxidant power and plant biomass decreased significantly [60].

Plant weight and chlorophyll content are interrelated parameters since chlorophyll is the key molecule that enables the generation of energy required for plant growth, so a reduction in chlorophyll content is often accompanied by a reduction in plant weight [62]. Our findings showed a reduction in both parameters at the concentrations of 300 and 400 mM NaCl. Consistently, also Mahmoudi et al. [63] reported a decrease in both plant growth and photosynthetic parameters in lettuce from the concentration of 200 mM NaCl. A similar trend was also observed by M'rah et al. [64] treating seedlings of Thellungiella halophila with NaCl concentrations in the range 0–200 mM and by Qin et al. [65] on lettuce and amaranth plants from NaCl concentration ranging 86–172 mM NaCl.

All FAAs analyzed, except for aspartic acid, showed increasing concentrations along with increasing NaCl. This trend has already been observed by Jouyban [66] and Gzik [67]. FAAs are important compounds in plant osmoregulation under salt and water stress [68]. An accumulation of FAAs reduces the osmotic potential in the cytoplasm and helps maintain water homeostasis between different cellular compartments [69].

Salinity caused an exponential increase in leaf Na accumulation. Treatment carried out with NaCl caused a significant reduction of leaf K from the concentration of 100 mM NaCl, likely due to the effect of ion selectivity [70,71]. This confirms that lettuce plants grown under salt stress can suffer from Na^+^ toxicity and K^+^ deficiency. Potassium performs several key roles in the activation of different enzymes, and it is known that Na^+^ cannot replace K^+^ in these roles [72]. For example, K^+^ plays a key role in protein synthesis, since it is essential for binding tRNA to ribosomes [73]. In this regard, our findings show that starting from the concentration of 200 mM NaCl the decrease in leaf K is paralleled by a significant reduction in leaf total soluble protein content.

### 4.2 Response following biochar addition

Our results showed that B addition did not make significant improvements in fresh weight but only a slightly positive trend at the concentrations of 300 and 400 mM NaCl. This ability of B to promote plant growth under salt stress conditions has been reported for tomato (*Solanum lycopersicum* L.) [33], corn (*Zea mays* L.) [34], and eggplant (*Solanum melongena* L.) [35]. It is well known that salt stress causes a reduction in chlorophyll content resulting in leaf chlorosis owing to damage to the photosynthetic machinery caused by the increase in chlorophyllase enzyme activity [74]. Our findings showed that plants grown with the addition of B have a significant increase in chlorophyll at the concentration of 400 mM NaCl. Similar results of B addition are reported for wheat (*Triticum aestivum* L.) [75,76], and common bean (*Phaseolus vulgaris* L.) [77].

Also of considerable importance is the significant increase in total antioxidant power at the concentrations of 300 and 400 mM on plants grown with B. The total antioxidant power, as measured by the DPPH method, is related to the free radical scavenging capacity, and thus an increase reflects a greater capacity of the plant to resist to oxidative stress [78]. Furthermore, our results showed that the addition of B brought the total antioxidant power content to the level of the controls in each treatment. This result from a nutritional point of view is of considerable importance given that an increase in this parameter in vegetables can have beneficial effects on human health e.g., against several cardiovascular diseases [79,80].

The increase in EL is considered a reliable index of damage induced by osmotic and ionic stress in plant tissues, since it indirectly measures the level of integrity and stability of cell plasma membranes [81]. A high soil salt level causes accumulation of electrolytes within plant cells [82], leading to the breakdown of plasma membranes and consequently to cell death [83]. Soil amendment with B significantly reduced EL accumulation. Our results are consistent with those by Parkash and Singh [35] for eggplant (*Solanum melongena* L.) and those by Al-Tabbal et al. [36] for kochia (*Bassia scoparia* L). Although not significant, the decrease in EL in the leaves of lettuce plants grown in B-amended soils suggests that biochar could limit salt uptake by plant roots, thus helping to alleviate osmotic and ionic stress in plants. However, in our case, this effect was not observed for Na content since no significant reduction of Na was evidenced in the leaves of B-treated plants compared to untreated plants. On the other hand, amending the soils with B did not affect the response of FAAs to NaCl addition but the plants, as the soil NaCl concentration increased, produced and accumulated FAAs probably to reduce the osmotic potential in the cytoplasm while trying to maintain water homeostasis between different cellular compartments [69].

Biochar failed to counteract Na accumulation in lettuce leaves, but there was a significant increase in K and Zn content at the concentrations of 200–400 mM and 400 mM, respectively. Potassium, as mentioned above, plays several fundamental roles in the reactions that occur within plants, so an increase in its concentrations due to B addition is of considerable importance. B can be a direct source of different nutrients relative to the feedstock and pyrolysis conditions used [84]. Recent studies [34,75,[85], [86], [87], [88], [89]] have shown that B-amendment of saline soils increases the soil nutrient content (Ca, Mg, K, N, and P) and indicated that the increase in K is the most important change since this element plays a key role for plant growth [75,[85], [86], [87], [88], [89]]. Recently, it has been reported that the application of B in saline soils has no effect on the concentration of exchangeable Na, Ca, and Mg but increased exchangeable K by 44% [89]. In our study, soil element concentrations were not investigated, but the increase in K concentrations in the leaves of lettuce plants suggests that B may have increased soil K bioavailability.

Three different mechanisms of action of biochar added to the soil in mitigating the negative effects of salt stress on wheat plants have recently been described: *i*) by binding Na^+^ to itself and thus reducing Na^+^ uptake by plants; *ii*) by increasing the concentration of potassium ion (K^+^) in the soil and consequently reducing Na^+^ uptake by plants since Na inhibits the uptake of K, and *iii*) by increasing the soil water content, which dilutes the Na^+^ concentration and consequently lowers its accumulation [75]. The efficacy of these action mechanisms has been confirmed at relatively low NaCl concentrations, ranging from 25 to 50 mM [75]. However, the effects of biochar in mitigating the effects of NaCl at concentrations >200 mM are unclear, as no study to date has been devoted to analyzing possible interactions between biochar and such high salt concentrations. Therefore, this study investigated the effects on lettuce plants of biochar addition to soils with high salt concentrations (specifically 300 and 400 mM NaCl). The results obtained lead us to speculate that biochar at 5% (w/w) in soil can have acted according to one or more of the proposed mechanisms in mitigating damage on lettuce plants up to concentration of 200 mM. This value could represent the threshold level beyond which the effectiveness of biochar is inhibited by the higher salt concentration in the soil. For this reason, since some results are not completely well understood, future studies testing the efficacy of using a biochar concentration >5% (w/w) in soils with NaCl levels >200 mM would be highly needed, given the increasing sea level rise over terrestrial lands and given the significant rise in the global average temperature level due to the current climate change phenomenon.

## 5 Conclusions

This study showed that the addition of 5% (w/w) biochar to the soil alleviates the negative effects of salt stress at high concentrations (i.e., 300 and 400 mM NaCl) by significantly increasing the total antioxidant power (at 300 mM: +67%; 400 mM: +167%) as well as the content of chlorophyll (at 400 mM: +167%), K (at 400 mM: +43%) and Zn (at 400 mM: +30%) in lettuce leaves. It also confirmed that low (i.e., 50 and 100 mM) NaCl concentrations did not damage lettuce plants but rather play a beneficial role as an eustressor by stimulating the plant growth.

Given the urgent need to find eco-friendly solutions with a view to environmental sustainability to the problem of soil salinity, the use of biochar could be a helpful agricultural practice to recover soil currently not cultivable.

## Funding

This research received no external funding.

## Data availability statement

Data included in article/supp. material/referenced in article.

## CRediT authorship contribution statement

**Riccardo Fedeli:** Writing – review & editing, Writing – original draft, Investigation, Data curation. **Andrea Vannini:** Writing – review & editing. **Nesrine Djatouf:** Investigation. **Silvia Celletti:** Writing – review & editing, Writing – original draft, Supervision, Methodology, Investigation, Data curation. **Stefano Loppi:** Writing – review & editing, Supervision, Resources, Data curation, Conceptualization.

## Declaration of competing interest

The authors declare that they have no known competing financial interests or personal relationships that could have appeared to influence the work reported in this paper.
