# Supplementary material for: Can lettuce plants grow in saline soils supplemented with biochar?
Source: Heliyon. 2024 Feb 15;10(4):e26526. doi: 10.1016/j.heliyon.2024.e26526 (PMC10884517; doi:10.1016/j.heliyon.2024.e26526)
Supplement: Multimedia component 1 [file mmc1.docx]

**SUPPLEMENTARY**

**Table S1**. Free amino acids (FAAs) of lettuce leaves expressed as median ± error. B0% = without biochar; B5% = with 5% (w/w) biochar. Different letters (lowercase for B0 and uppercase for B5) indicate significant differences between the same treatment; where letters are not present, there is no statistical difference; n.d. = not determined.

| **FAAs**  **(µg g_FW_^-1^)** | **0** | | **50** | | **100** | | **200** | | **300** | | **400** | | **NaCl [mM]** |
| --- | --- | --- | --- | --- | --- | --- | --- | --- | --- | --- | --- | --- | --- |
|  | **0** | **5** | **0** | **5** | **0** | **5** | **0** | **5** | **0** | **5** | **0** | **5** | **B (%)** |
| **Ala** | 15.41 ± 0.73^b^ | 10.32 ±  4.85 | 19.33 ± 4.38^ab^ | 21.32 ± 4.50 | 26.38 ± 1.78^ab^ | 18.04 ± 2.60 | 28.48 ± 1.23^a^ | 21.54 ± 3.37 | 25.81 ± 4.47^ab^ | 26.71 ± 2.29 | 31.44 ± 4.79^ab^ | 28.98 ± 9.91 |  |
| **Asp** | 20.13 ± 1.99 | 13.91 ±  0.15 | 24.61 ± 7.45 | 19.36 ± 2.41 | 27.33 ± 1.97 | 16.74 ± 1.47 | 32.23 ± 4.28 | 20.08 ± 3.38 | 22.69 ± 5.50 | 21.97 ± 1.99 | 21.45 ± 5.87 | 23.73 ± 6.72 |  |
| **Glu** | 38.22 ± 5.39^b^ | 29.82 ±  4.45 | 58.01 ± 6.12^ab^ | 73.90 ± 7.85 | 75.21 ± 4.54^a^ | 43.43 ± 3.11 | 53.73 ± 5.68^ab^ | 39.26 ± 12.44 | 72.50 ± 9.19^ab^ | 48.43 ± 3.04 | 89.45 ± 11.51^a^ | 52.67 ± 14.07 |  |
